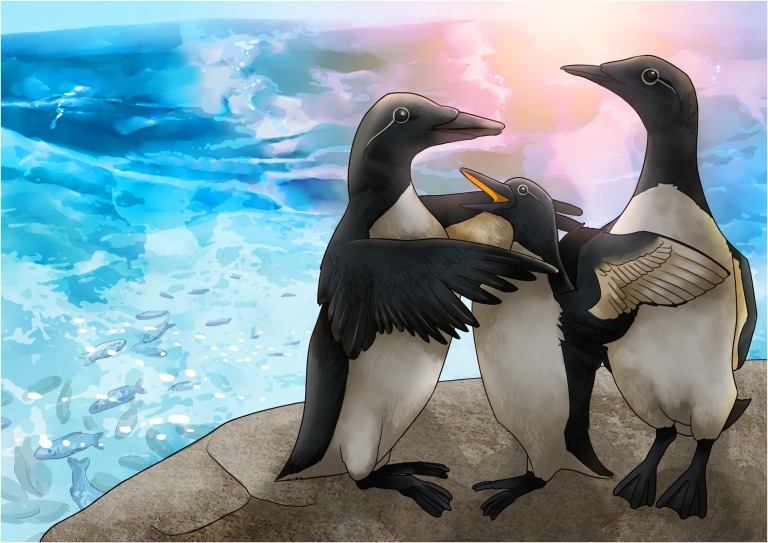# Prudent parenting: murre now or murre later?

**DOI:** 10.1093/conphys/cox071

**Published:** 2017-12-08

**Authors:** Phillipa Beale

**Affiliations:** Australian National University, Research School of Biology, 134 Linnaeus Way, Acton ACT 2601, Australia; Ecology and Evolution Division, Research School of Biology, 134 Linnaeus Way, Acton ACT, 2601, Australia

Diving into parenthood is never easy, but for common murres (*Uria aalge*) some positively prudent parenting is required. These birds have to decide whether they care more about the chicks they have got or about having additional chicks in the future. Anne Storey and her colleagues were able to measure the physiological tell-tale signs of this balancing act both when food is scarce and when food is abundant. And, the team showed how stressing less and diving deep can help when raising chicks (Storey *et al.*, 2017).

Murres are a long-lived seabird that forages on the capelin fish. When capelin are abundant, life is good. Murres catch the fish, feed their chicks, and everyone is fat and happy. Interestingly, murres make some clear adjustments to this protocol when capelins are less abundant. Storey and her team measured hormones that indicate stress and fat breakdown and the blood's oxygen carrying capacity. Together, with changes in body mass of the murres, the team could paint a better picture of how the birds are dealing with parenthood during slim times.

What balance did the Murres strike? The most surprising result is that stress hormone levels were highest during years when capelins were in medium abundance. One might expect that lower food supplies result in more stress hormones. However, stress hormones drive the birds to forage more. When food is abundant, there is no need for that, but when food is scarce, the return for so much effort is small, making high-stress hormone levels disadvantageous. When the murres have high levels of stress hormones, like those with newly hatched chicks, they lose body mass. Not surprisingly, birds that maintained low levels of stress hormones tended to do better in the long run. But could lower stress be because another food-fish species becomes more abundant when capelin are scarce? It is possible, but researchers cannot say quite yet.

That's where the blood's carrying capacity for oxygen comes into play. Unfortunately for the murres, capelin do not leap from the ocean into the nest, offering themselves as a tasty snack. Instead, the murres have to dive into the water to retrieve them. When there are fewer fish around, this means murres are diving deeper, longer, and more frequently. Having a higher blood oxygen carrying capacity and being slim makes this easier.

So, murres are able to adjust their investment in their chicks to match the availability of capelin so that they do not get exhausted. This way, they can maintain their own health to successfully rear chicks in the future. It is the opposite of a live fast, die young YOLO-type of attitude. If birds drove cars, a murre would drive a Prius, not a Porsche.

These results also tell us that we can use physiological measurements to get an idea of how murres are coping with the costs of breeding, which can change seasonally, and how hormones and body mass play into this. It turns out that to succeed, just be big, be relaxed, and be a really prudent parent… murre or less.

Illustration by Erin Walsh; Email: ewalsh.sci@gmail.com

**Figure:**